# Procalcitonin and pyuria-based algorithm reduces antibiotic use in urinary tract infections: a randomized controlled trial

**DOI:** 10.1186/s12916-015-0347-y

**Published:** 2015-05-01

**Authors:** Daniel Drozdov, Stefanie Schwarz, Alexander Kutz, Eva Grolimund, Anna Christina Rast, Deborah Steiner, Katharina Regez, Ursula Schild, Merih Guglielmetti, Antoinette Conca, Barbara Reutlinger, Cornelia Ottiger, Florian Buchkremer, Sebastian Haubitz, Claudine Blum, Andreas Huber, Ulrich Buergi, Philipp Schuetz, Andreas Bock, Christoph Andreas Fux, Beat Mueller, Werner Christian Albrich

**Affiliations:** Medical University Department, University of Basel, Kantonsspital Aarau, Tellstrasse, Aarau, 5001 Switzerland; Department of Clinical Nursing Science, Kantonsspital Aarau, Tellstrasse, Aarau, 5001 Switzerland; Department of Laboratory Medicine, Kantonsspital Aarau, Tellstrasse, Aarau, 5001 Switzerland; Division of Nephrology, Kantonsspital Aarau, Tellstrasse, Aarau, 5001 Switzerland; Department of Emergency Medicine, Kantonsspital Aarau, Tellstrasse, Aarau, 5001 Switzerland; Division of Infectious Diseases, Kantonsspital Aarau, Tellstrasse, Aarau, 5001 Switzerland; Department of Infectious Diseases and Hospital Epidemiology, Kantonsspital St. Gallen, Rorschacherstrasse 95, St. Gallen, 9007 Switzerland

**Keywords:** Antibiotic stewardship, Biomarker, Procalcitonin, Urinary tract infection

## Abstract

**Background:**

Urinary tract infections (UTIs) are common drivers of antibiotic use. The minimal effective duration of antibiotic therapy for UTIs is unknown, but any reduction is important to diminish selection pressure for antibiotic resistance, costs, and drug-related side-effects. The aim of this study was to investigate whether an algorithm based on procalcitonin (PCT) and quantitative pyuria reduces antibiotic exposure.

**Methods:**

From April 2012 to March 2014, we conducted a factorial design randomized controlled open-label trial. Immunocompetent adults with community-acquired non-catheter-related UTI were enrolled in the emergency department of a tertiary-care 600-bed hospital in northwestern Switzerland. Clinical presentation was used to guide initiation and duration of antibiotic therapy according to current guidelines (control group) or with a PCT-pyuria-based algorithm (PCT-pyuria group).

The primary endpoint was overall antibiotic exposure within 90 days. Secondary endpoints included duration of the initial antibiotic therapy, persistent infection 7 days after end of therapy and 30 days after enrollment, recurrence and rehospitalizations within 90 days.

**Results:**

Overall, 394 patients were screened, 228 met predefined exclusion criteria, 30 declined to participate, and 11 were not eligible. Of these, 125 (76% women) were enrolled in the intention-to-treat (ITT) analysis and 96 patients with microbiologically confirmed UTI constituted the per protocol group; 84 of 125 (67%) patients had a febrile UTI, 28 (22%) had bacteremia, 5 (4%) died, and 3 (2%) were lost to follow-up. Overall antibiotic exposure within 90 days was shorter in the PCT-pyuria group than in the control group (median 7.0 [IQR, 5.0–14.0] vs. 10.0 [IQR, 7.0–16.0] days, *P* = 0.011) in the ITT analysis. Mortality, rates of persistent infections, recurrences, and rehospitalizations were not different.

**Conclusions:**

A PCT-pyuria-based algorithm reduced antibiotic exposure by 30% when compared to current guidelines without apparent negative effects on clinical outcomes.

**Trial registration:**

Current controlled trials ISRCTN13663741, date applied: 22/05/2012, date assigned: 03/07/2012, last edited: 28/01/2014.

**Electronic supplementary material:**

The online version of this article (doi:10.1186/s12916-015-0347-y) contains supplementary material, which is available to authorized users.

## Background

Urinary tract infections (UTIs) are common drivers of antibiotic use and hospitalizations. In the United States, almost 10 million outpatients are diagnosed with UTIs each year [1], and UTIs account for 16% of all infectious disease-related hospitalizations and 6% of all infectious disease-related deaths [2]. Despite various prevention strategies, recurrent UTIs are very common [3].

Any reduction of antibiotic exposure is important to diminish selection pressure for antibiotic resistance, costs, and drug-related side-effects [4]. Current guidelines for antibiotic treatment duration of febrile UTI/pyelonephritis largely reflect expert opinion [5-8], as only few intervention studies compared different durations [9-13]. Most studies in this regard were performed to establish short-term therapy in uncomplicated simple UTIs [14,15]. Motivated by the emerging antibiotic resistance of uropathogens [16-18] and the ecological effects of antibiotics, the minimal effective duration of antibiotic therapy is being challenged [19], especially in the elderly, in whom UTIs are often over diagnosed and therefore over treated. The use of biomarkers might improve the management of patients with UTIs [20].

The biomarker procalcitonin (PCT) was established for antibiotic stewardship in respiratory tract infections [21,22] and sepsis [23]. As an indicator for systemic infections [24], it showed high prognostic value to predict bacteremia in patients with urosepsis [25]. In the PRORATA trial, which documented a safe reduction of antibiotic usage through a PCT-guided strategy in patients with sepsis, UTIs were the source of infection in 7% of patients [26]. In patients with lower UTIs, PCT may need to be complemented with inflammatory surrogates of local infection such as pyuria. Normalization of pyuria in the first days after initiation of therapy correlated with a successful outcome in women with lower UTIs [27]. Therefore, we assumed that an algorithm based on PCT and pyuria could safely reduce the duration of antibiotic therapy in patients with UTIs.

## Methods

### Design overview

We conducted an investigator-initiated, single-center, factorial design, randomized controlled (1:1) and open-label trial. Herein, we focus on the efficacy of an algorithm based on clinical symptoms, PCT, and pyuria to safely reduce the duration of antibiotic therapy compared to current guidelines [6,15,28]. Details of the full trial design and methods have been published elsewhere [29].

The trial was conducted in accordance with the ethical principles of the Helsinki Declaration. The local ethical review committee of the Canton Aargau, Switzerland, approved the study protocol. All patients or their next-of-kin provided written informed consent.

### Setting and participants

From April 2012 to March 2014, we screened all consecutive immunocompetent adults with community-acquired non-catheter-related UTIs as the main diagnosis presenting to the emergency department of the cantonal hospital of Aarau, a tertiary-care 600-bed hospital in northwestern Switzerland.

A UTI was defined by at least one clinical symptom (temperature ≥38.0°C, urinary urgency, dysuria, suprapubic pain, flank pain, costovertebral angle tenderness, nausea, and vomiting) and one urinary criterion (pyuria >20 leukocytes/μL, obtained by flow cytometry UF1000i (Sysmex) [27,30], and/or evidence of nitrite). Presence of flank pain, costovertebral angle tenderness, and/or a body temperature ≥38.0°C defined a ‘febrile UTI/pyelonephritis’; otherwise, patients were considered to have a ‘simple UTI’. The criteria for a ‘complicated UTI’ included any patient of at least 70 years, male gender, duration of symptoms of more than 7 days, previous antibiotic therapy within 30 days, at least two prior UTIs in the last 6 months or at least three during the last 12 months, any urologic intervention within 30 days, functional or anatomic abnormality, diabetes mellitus, or immunosuppressive therapy; otherwise it was referred to as an ‘uncomplicated UTI’.

Patients were excluded if they presented with other infections that required antibiotic therapy or had been treated with antibiotics within 48 hours before presentation; pregnancy; prostatitis defined as painful digital rectal examination, a prostate-specific antigen value of >4 ng/mL or ≥2× baseline before infection; foreign bodies within the urinary tract; endovascular prostheses; non-endovascular prostheses within 6 months after implantation; insufficient language skills with no possibility for translation; foreseeable non-compliance for follow-up, e.g., current drug abuse; severe immunodeficiency: neutrophils <500/μL, CD4 cells <350/μL in patients with HIV-infection, leukemia, lymphoma, myeloma, cytotoxic medications, hemodialysis, transplant patients; or life-threatening medical comorbidities leading to possible imminent death.

### Randomization and interventions

The allocation of patients to either the PCT-pyuria group or the control group was based on a pre-specified computer generated randomization list and was concealed on the study website.

**Figure 1:**
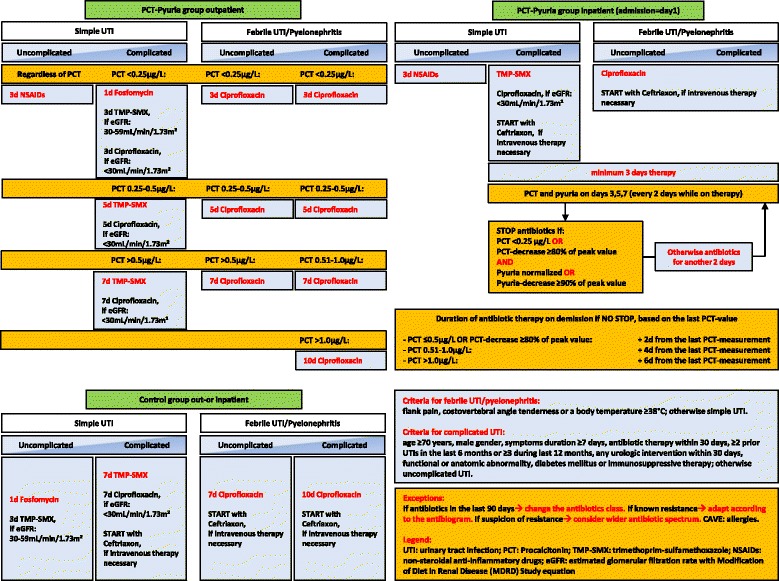
Algorithm for procalcitonin (PCT) and pyuria-guided therapy. TMP-SMX, Trimethoprim-sulfamethoxazole; NSAIDs, Non-steroidal anti-inflammatory drugs; eGFR, Estimated glomerular filtration rate with Modification of Diet in Renal Disease Study equation.

Clinical presentation without (control group) or with PCT and pyuria (PCT-pyuria group) was used to guide initiation and duration of antibiotic therapy. The choice of antibiotics and the minimal duration of therapy were based on recent guidelines [6,15]. The algorithm is presented in Figure 1.

For simple UTIs, we used fosfomycin (3 g single dose) [31], trimethoprim-sulfamethoxazole (800/160 mg twice daily) for estimated glomerular filtration rates (eGFR) 30 to 59 mL/min/1.73 m^2^ or ciprofloxacin (250 mg twice daily) for eGFR <30 mL/min/1.73 m^2^. In the PCT-pyuria group, patients with uncomplicated simple UTIs were planned to receive only non-steroidal anti-inflammatory drugs (NSAIDs) for 3 days regardless of PCT-values [32,33].

For febrile UTIs/pyelonephritis, ciprofloxacin was chosen as standard oral treatment for better comparability with recent studies (500 mg orally twice daily, respectively ciprofloxacin 250 mg orally twice daily for eGFR <30 mL/min/1.73 m^2^), if intravenous therapy was necessary ceftriaxone (2 g once daily) was chosen. When known, antibiotic resistance profiles of prior or current pathogens were taken into account and antibiotic therapy was adjusted.

In inpatients in the PCT-pyuria group, antibiotic duration was based on absolute PCT-values and relative decreases of PCT-levels, as well as pyuria. PCT and pyuria were measured on admission in all patients; in hospitalized patients, the measurements were continued every other day till the end of antibiotic therapy. For outpatients in the PCT-pyuria group, antibiotic duration was calculated according to absolute values of PCT at baseline. In the control group, antibiotic therapy durations were recommended based on current guidelines [6,15] (Figure 1).

Urinalysis and urine culture with an antibiotic resistance profile were performed on admission. In patients with febrile UTI, blood cultures were taken prior to administration of antibiotics.

To determine microbiological cure and recurrence rates urinalysis and urine culture were performed on day 7 after end of therapy and day 30 after enrollment. Patients were instructed in collection of midstream urine for urinalysis and urine culture. These urine specimens were sent by mail and reached the laboratory within 48 hours.

### Outcomes and follow-up

All endpoints were assessed at discharge from hospital and 30 and 90 days after enrollment through standardized telephone interviews by blinded members of the study team.

The primary endpoint was overall antibiotic exposure within 90 days. Each day of antibiotic therapy for any indication was counted as a full day of antibiotic exposure.

Secondary endpoints included duration of the initial antibiotic therapy; overall rate of recurrence, defined as any re-treated UTI; overall rate of rehospitalization for any cause within 90 days after enrollment; rate of persistent infection 7 days after end of therapy, defined as recovery of the initial uropathogen in the returned urine culture plus pyuria >20 leucocytes/μL in the concomitant urinalysis; and rate of persistent symptomatic infection 30 days after enrollment, defined as recovery of the initial uropathogen in the returned urine culture plus pyuria >20 leucocytes/μL in the concomitant urinalysis plus symptoms of an UTI in the 30 days phone interview.

All patients with positive urine cultures and pyuria in the follow-up samples were contacted by phone by members of the study team and advised to visit a general practitioner or an emergency room. The laboratory results were sent to the primary care providers when available, but no further guidance was given as to whether to prescribe an antibiotic.

Any serious adverse event, rehospitalization for any cause, recurrence, or death of any cause within 90 days after enrollment were monitored by the data safety and monitoring board, which consisted of three independent experts in infectious diseases, nephrology, and epidemiology.

### Statistical analysis

The sample size was calculated assuming that duration of antibiotic therapy would be two days shorter in the PCT-pyuria group (8 days, standard deviation ±5) than in the control group (10 days, standard deviation ±5). Accordingly, 99 patients per arm would provide an 80% power at the 5% alpha level [29].

The primary analysis was performed including all randomized patients following an intention-to-treat (ITT) principle. The per-protocol (PP) analysis was performed in a defined population with microbiologically confirmed UTI and without patients who violated inclusion or exclusion criteria or were lost to follow-up. If the initial urine culture was missing, sterile, or contaminated but a uropathogen was isolated in concomitantly withdrawn blood cultures, the patients were still included in the PP analysis. We used predefined urine culture cut-offs for significance [29].

As the study was conceived in a two-by-two factorial design, we checked for interaction between the two randomization arms. As there was no evidence for interaction, the second randomization (proadrenomedullin-assisted site of care decision [29]) was no longer considered for this analysis.

Discrete variables were expressed as counts (percentage); continuous variables as medians and interquartile range, unless stated otherwise. For the analysis of the primary endpoint of antibiotic exposure, we used the Mann–Whitney U test for similarly shaped not normally distributed continuous data. In sensitivity analysis, we also used median regression to model the primary endpoint and adjusted the analysis for bacteremia. Logistic regression analyses of persistent infection, recurrence, and rehospitalization rates were performed to assess safety endpoints. Since this was a feasibility study we did not plan to have enough power for non-inferiority testing.

## Results

**Figure 2:**
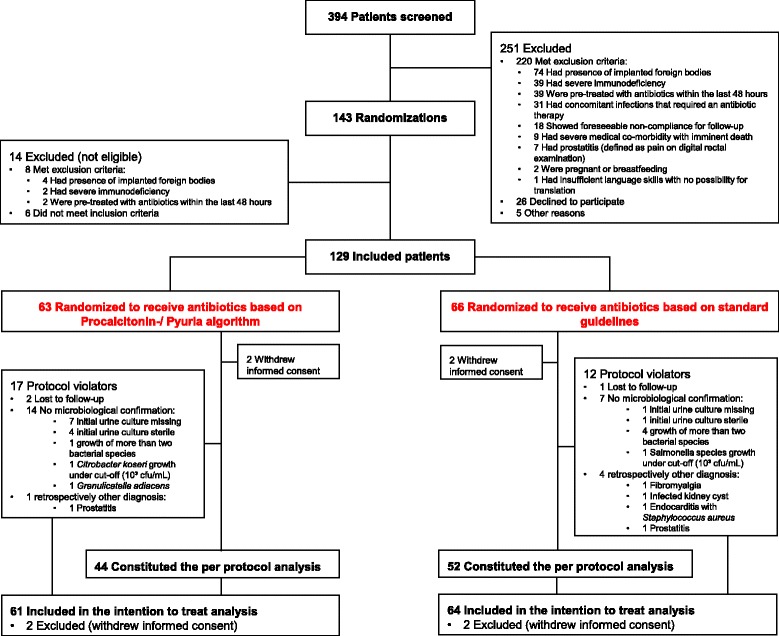
Flow diagram of patients in trial.

We screened 394 patients, of whom 269 were not eligible due to the exclusion criteria, patient refusal, or other reasons. Thus, 125 patients were enrolled in the ITT analysis and 3 (2%) patients were lost to follow-up. As predefined, 96 (77%) patients with microbiologically confirmed UTIs and no protocol violations constituted the PP analysis (Figure 2).

In five patients, the final diagnosis was retrospectively different from a UTI: one patient with an infected kidney cyst with *Klebsiella oxytoca* in blood cultures and two patients with prostatitis with *Staphylococcus aureus* in blood and urine cultures and with *Escherichia coli* in urine culture, respectively, received prolonged courses of antibiotics. One patient with severe sepsis due to *Staphylococcus aureus* endocarditis died during the initial hospitalization, and another patient was diagnosed with fibromyalgia syndrome as explanation for costovertebral angle tenderness and had a sterile urine culture.

The baseline characteristics were generally well-balanced except for the distribution of patients with bacteremia, namely 19 (30%) in the control group and 9 (15%) in the PCT-pyuria group (*P* = 0.045; Table 1); 95 (76%) of the 125 patients were women and 84 (67%) had a febrile UTI. The median age was 73 years (range, 19–96 years); 35 (28%) were treated as outpatients.

**Table 1:** **Baseline characteristics by randomization group**

	**Control group**	**PCT/Pyuria group**	***P*** **value** ^**a**^
**n**	64	61	
**Demographics**			
Age, median (IQR), y	75 (51–80)	71 (44–81)	0.65
Females, n (%)	52 (81%)	43 (70%)	0.16
**Charlson comorbidity index, median (IQR)**	4 (1-7)	3 (0-6)	0.20
**Clinical history, n (%)**			
Dysuria	27 (42%)	29 (48%)	0.55
Urinary urgency	27 (43%)	22 (36%)	0.44
Frequent urination	23 (36%)	24 (39%)	0.69
Flank pain	20 (31%)	21 (34%)	0.71
**Clinical findings, median (IQR)**			
Confusion, n (%)	16 (25%)	13 (21%)	0.63
Body temperature, °C	37.7 (36.9–38.6)	38.2 (37.0–39.0)	0.20
Systolic blood pressure, mm Hg	128 (110–145)	120 (108–135)	0.055
Diastolic blood pressure, mm Hg	69 (60–80)	70 (60–79)	0.85
Respiratory rate, breaths/min	16 (15–20)	16 (14–18)	0.18
**Laboratory findings, median (IQR)**			
Leukocyte count, × 10^9^cells/L	10.77 (7.83–14.34)	12.28 (9.18–15.65)	0.12
C-reactive protein, mg/L	34 (9–142)	44 (7–131)	0.84
PCT, μg/L	0.20 (0.08–1.34)	0.32 (0.11–1.32)	0.68
PCT ≥0.25 μg/L, n (%)	29 (45%)	33 (54%)	0.33
Serum creatinine, μmol/L	100 (77–136)	93 (74–117)	0.60
Blood urea nitrogen, mmol/L	6.5 (4.9–10.2)	6.3 (4.6–9.3)	0.64
**Bacteremic patients, n (%)**	19 (30%)	9 (15%)	**0.045**
**Final diagnosis, n (%)**			
Uncomplicated simple UTI	6 (9%)	2 (3%)	0.36
Complicated simple UTI	12 (19%)	16 (26%)	
Uncomplicated febrile UTI/pyelonephritis	8 (13%)	9 (15%)	
Complicated febrile UTI/pyelonephritis	34 (53%)	33 (54%)	
Other final diagnosis^b^	4 (6%)	1 (2%)	
**Complicated UTI**	46 (72%)	49 (80%)	0.27
Male patients	12 (19%)	18 (30%)	0.16
Patients older than or equal 70 years	35 (55%)	34 (56%)	0.91
Patients with symptoms longer than 7 days	10 (16%)	7 (12%)	0.50
Patients with antibiotic therapy in the last 30 days	5 (8%)	10 (16%)	0.14
Patients with diabetes	16 (25%)	8 (13%)	0.092
Patients with recurrent UTIs	5 (8%)	9 (15%)	0.22
Patients with urologic interventions in the last 30 days	3 (5%)	2 (3%)	0.69
Patients with anatomic abnormalities	2 (3%)	5 (8%)	0.22
Patients with immunosuppression	0	2 (3%)	0.14
**Patients from long-term healthcare facilities**	5 (8%)	4 (7%)	0.79
**Hospitalized patients, n (%)**	45 (70%)	45 (74%)	0.67

^a^χ^2^ test for categorical variables, 2-sample *t*-test for continuous variables.

^b^Prostatitis (n = 2), infected kidney cyst (n = 1), endocarditis (n = 1), fibromyalgia syndrome (n = 1).

IQR, Interquartile range; y, Years.

*Escherichia coli* was identified in 72% of urine and blood culture isolates (Table 2). Ciprofloxacin and trimethoprim-sulfamethoxazole resistance in *E. coli* were 12% and 22%, respectively. In the PP analysis, the first-line antibiotic therapy was efficacious in 44% to 100%, as presented in Table 3.

**Table 2:** **Microbiological results at baseline**

**Isolation of uropathogens in baseline urine culture**		
**n (%)**	**Control group**	**PCT/Pyuria group**
**All patients (intention-to-treat)**	**(n = 64)**	**(n = 61)**
Missing urine cultures	2 (3%)	8 (13%)
Sterile urine cultures	4 (6%)	4 (7%)
Contaminated urine cultures	6 (9%)	3 (5%)
Urine cultures with bacterial growth	52 (82%)	46 (75%)
**Total no. of isolates** ^**a**^	53	52
**Gram-negative uropathogen**		
*Escherichia coli*	37/53 (70%)	39/52 (75%)
*Klebsiella pneumoniae*	6/53 (11%)	3/52 (6%)
*Citrobacter koseri*	0	1/52 (2%)
*Pseudomonas aeruginosa*	0	1/52 (2%)
*Proteus mirabilis*	0	1/52 (2%)
**Gram-positive uropathogen**		
*Enterococcus faecalis*	3/53 (6%)	6/52 (12%)
*Aerococcus urinae*	1/53 (2%)	0
**Non uropathogen**		
*Staphylococcus aureus*	3/53 (6%)	0
Lactobacillus species^b^	2/53 (4%)	0
*Granulicatella adiacens*	0	1/52 (2%)
Salmonella species	1/53 (2%)	0
**Isolation of uropathogens in baseline blood cultures**		
**n (%)**	**Control group**	**PCT/Pyuria group**
**All patients (intention-to-treat)**	**(n = 64)**	**(n = 61)**
**Blood samples for culture obtained**	44 (69%)	44 (72%)
**No. (%) of contaminated blood cultures**	2/44 (5%)	2/44 (5%)
Coagulase-negative staphylococci	2/44 (5%)	1/44 (2%)
*Propionibacterium acnes*	0	1/44 (2%)
**No. (%) of positive blood cultures**	19/44 (43%)	9/44 (20%)
**Gram-negative uropathogen**		
*Escherichia coli*	14/19 (74%)	9/9 (100%)
*Klebsiella oxytoca*	1/19 (5%)^c^	0
**Gram-positive uropathogen**		
*Enterococcus faecalis*	1/19 (5%)	0
**Non uropathogen**		
*Staphylococcus aureus*	2/19 (11%)^c^	0
Lactobacillus species	1/19 (5%)	0

^a^Two isolates in five cultures, three isolates in one culture.

^b^Lactobacillus species was not a classical uropathogen but in one case confirmed by concomitant positive blood cultures with Lactobacillus species (4/4 samples).

^c^Three patients not included in the per protocol analysis with final diagnosis other than UTI: *Klebsiella oxytoca* in one patient with infected kidney cyst, *Staphylococcus aureus* in one patient with endocarditis, and one patient with prostatitis.

**Table 3:** **Initial antibiotic therapy by randomization group**

	**Control group**	**PCT/Pyuria group**
**All patients (intention-to-treat)**	**(n = 64)**	**(n = 61)**
**First-line antibiotic therapy**		
Ciprofloxacin	25 (39%)	20 (33%)
Ceftriaxon	18 (28%)	18 (30%)
TMP-SMZ	9 (14%)	15 (25%)
Fosfomycin	6 (9%)	3 (5%)
Amoxicillin-Clavulanate	5 (8%)	1 (2%)
Other	1 (2%)	3 (5%)
NSAIDS	0	1 (2%)
**Second-line antibiotic therapy**		
Ciprofloxacin	10 (16%)	15 (25%)
Ceftriaxon	4 (6%)	4 (7%)
TMP-SMZ	4 (6%)	3 (5%)
Amoxicillin-Clavulanate	4 (6%)	3 (5%)
Other	3 (5%)	5 (8%)
**Per-protocol population**	**(n = 52)**	**(n = 44)**
**First-line antibiotic therapy**		
Ciprofloxacin	20 (38%), efficacious in 90%	15 (34%), efficacious in 87%
Ceftriaxon	17 (33%), efficacious in 76%	14 (32%), efficacious in 64%
TMP-SMZ	6 (12%), efficacious in 67%	9 (20%), efficacious in 44%
Fosfomycin	5 (10%), efficacious in 100%	2 (5%), efficacious in 100%
Amoxicillin-Clavulanate	3 (6%), efficacious in 67%	1 (2%), efficacious in 100%
Other	1 (2%)	2 (4%)
NSAIDS	0	1 (2%)
**Second-line antibiotic therapy**		
Ciprofloxacin	10 (19%), efficacious in 100%	11 (25%), efficacious in 82%
Ceftriaxon	3 (6%), efficacious in 67%	4 (9%), efficacious in 100%
TMP-SMZ	3 (6%), efficacious in 100%	3 (7%), efficacious in 100%
Amoxicillin-Clavulanate	4 (8%), efficacious in 100%	2 (5%), efficacious in 100%
Other	5 (10%)	3 (7%)

TMP-SMX, Trimethoprim-sulfamethoxazole; NSAIDs, Non-steroidal anti-inflammatory drugs.

### Primary endpoint

The overall antibiotic exposure within 90 days was shorter in the PCT-pyuria group, both in the ITT analysis (7.0 vs. 10.0 days, *P* = 0.011) and in the PP analysis (7.0 vs. 10.0 days, *P* = 0.025) groups. In subgroup analyses, PCT-pyuria led to shorter antibiotic therapies in women (7.0 vs. 10.0 days, *P* = 0.022) and in patients with complicated febrile UTI/pyelonephritis (7.5 vs. 11.0 days, *P* = 0.002); for male patients, the difference did not reach statistical significance (9.0 vs. 16.0 days, *P* = 0.062; Table 4). The statistical analysis for predefined subgroups uncomplicated simple UTI, complicated simple UTI, and uncomplicated febrile UTI/pyelonephritis was limited by small numbers.

**Table 4:** **Antibiotic exposure by randomization group with subgroup analysis**

	**Control group**	**PCT/Pyuria group**	***P*** **value** ^**a**^
	**Median (IQR), days**	**Median (IQR), days**	
**All patients (intention-to-treat)**	**(n = 64)**	**(n = 61)**	
Duration of initial antibiotic therapy	10.0 (7.0–11.0)	6.0 (4.0–8.0)	**<0.001**
Antibiotic exposure within 90 days	10.0 (7.0–16.0)	7.0 (5.0–14.0)	**0.011**
**Per-protocol population**	**(n = 52)**	**(n = 44)**	
Duration of initial antibiotic therapy	10.0 (7.0–11.0)	6.0 (4.0–7.0)	**<0.001**
Antibiotic exposure within 90 days	10.0 (7.5–16.0)	7.0 (5.0–14.5)	**0.025**
**Female patients**	**(n = 52)**	**(n = 43)**	
Duration of initial antibiotic therapy	9.0 (7.0–10.5)	5.0 (4.0–7.0)	**<0.001**
Antibiotic exposure within 90 days	10.0 (7.0–14.0)	7.0 (4.0–13.0)	**0.022**
**Male patients**	**(n = 12)**	**(n = 18)**	
Duration of initial antibiotic therapy	11.0 (10.5–12.0)	6.5 (5.0–9.0)	**0.003**
Antibiotic exposure within 90 days	16.0 (11.0–21.0)	9.0 (6.0–19.0)	0.062
**Inpatients**	**(n = 45)**	**(n = 45)**	
Duration of initial antibiotic therapy	10.0 (8.0–11.0)	7.0 (5.0–9.0)	**<0.001**
Antibiotic exposure within 90 days	11.0 (10.0–18.5)	8.5 (6.5–16.5)	**0.023**
**Uncomplicated simple UTI**	**(n = 6)**	**(n = 2)**	
Duration of initial antibiotic therapy	1.0 (1.0–1.0)	0.5 (0.0–1.0)	0.127
Antibiotic exposure within 90 days	1.0 (1.0–1.0)	4.0 (1.0–7.0)	0.513
**Complicated simple UTI**	**(n = 12)**	**(n = 16)**	
Duration of initial antibiotic therapy	7.0 (7.0–9.0)	4.0 (2.5–5.5)	**0.005**
Antibiotic exposure within 90 days	10.0 (8.0–12.0)	5.5 (3.0–14.0)	0.083
**Uncomplicated febrile UTI/pyelonephritis**	**(n = 8)**	**(n = 9)**	
Duration of initial antibiotic therapy	7.0 (7.0–7.5)	4.0 (4.0–6.0)	**0.009**
Antibiotic exposure within 90 days	7.0 (7.0–10.5)	6.5 (4.0–11.0)	0.238
**Complicated febrile UTI/pyelonephritis**	**(n = 34)**	**(n = 33)**	
Duration of initial antibiotic therapy	10.5 (10.0–11.0)	7.0 (6.0–9.0)	**<0.001**
Antibiotic exposure within 90 days	11.0 (10.0–18.0)	7.5 (6.5–13.5)	**0.002**

^a^Mann–Whitney U*-*test.

IQR, Interquartile range.

### Secondary endpoint

The initial antibiotic therapy was shorter in the PCT-pyuria group both in the ITT and PP analysis. This applies to all subgroup analyses except for patients with uncomplicated simple UTI (n = 8; Table 4).

### Safety outcomes

The clinical recurrence rate and rehospitalization rates within 90 days were similar in both groups (Table 5). Overall, five (4%) patients died, one in the PCT-pyuria group and four in the control group (Table 5). Safety outcomes were similar between the PCT-pyuria and the control group also within the subgroups of uncomplicated simple UTI, complicated simple UTI, uncomplicated febrile UTI/pyelonephritis, and complicated febrile UTI/pyelonephritis (Table 5).

**Table 5:** **Rates of adverse outcomes**

	**Control group**	**PCT/Pyuria group**	**Odds ratio**	**95% CI**	***P*** **value** ^**a**^
**All patients (intention-to-treat)**	**(n = 64)**	**(n = 61)**			
Recurrence	14/63 (22%)	15/59 (25%)	1.19	0.52–2.75	0.68
Rehospitalization	17/63 (27%)	15/59 (25%)	0.92	0.41–2.07	0.85
Death	4/63 (6%)	1/59 (2%)	0.25	0.03–2.30	0.22
**Per-protocol population**	**(n = 52)**	**(n = 44)**			
Recurrence	11/52 (21%)	14/44 (32%)	1.74	0.69–4.36	0.24
Rehospitalization	13/52 (35%)	13/44 (41%)	1.26	0.51–3.10	0.62
Death	1/52 (2%)	0			
**Inpatients**	**(n = 45)**	**(n = 45)**			
Recurrence	11 (25%)	13 (30%)	1.26	0.49–3.22	0.63
Rehospitalization	15 (34%)	13 (30%)	0.81	0.28–0.96	0.65
**Uncomplicated simple UTI**	**(n = 6)**	**(n = 2)**			
Recurrence	1/6 (17%)	1/2 (50%)	5.00	0.15–166.59	0.37
Rehospitalization	1/6 (17%)	1/2 (50%)	5.00	0.15–166.59	0.37
**Complicated simple UTI**	**(n = 12)**	**(n = 16)**			
Recurrence	3/11 (27%)	6/16 (38%)	1.60	0.30–8.49	0.58
Rehospitalization	2/11 (18%)	6/16 (38%)	2.70	0.43–16.94	0.29
**Uncomplicated febrile UTI/pyelonephritis**	**(n = 8)**	**(n = 9)**			
Recurrence	1/8 (13%)	2/8 (25%)	2.33	0.17–32.58	0.53
Rehospitalization	2/8 (25%)	1/8 (13%)	0.43	0.03–5.98	0.53
**Complicated febrile UTI/pyelonephritis**	**(n = 34)**	**(n = 33)**			
Recurrence	8/34 (24%)	6/32 (19%)	0.75	0.23–2.47	0.64
Rehospitalization	11/34 (32%)	7/32 (22%)	0.59	0.19–1.77	0.34

^a^Logistic regression analysis.

The rate of persistent infection (6% in the control group vs. 6% in the PCT-pyuria group in ITT analysis) and of new infections 7 days after end of therapy were similar in both groups (6% in the control group vs. 6% in the PCT-pyuria group in ITT analysis). The microbiological isolates obtained from the urine cultures on day 7 after end of therapy are presented in Table 6.

**Table 6:** **Microbiological outcome 7 days after end of therapy**

**n (%)**	**Control group**	**PCT/Pyuria group**	**Odds ratio**	**95% CI**	***P*** **value** ^**a**^
**All patients (intention-to-treat)**	**(n = 64)**	**(n = 61)**			
**Missing urinalysis**	10/64 (16%)	8/61 (13%)	0.82	0.30–2.22	0.69
**Missing urine cultures**	8/64 (13%)	8/61 (13%)	1.06	0.37–3.02	0.92
**Sterile urine cultures**	19/56 (34%)	20/53 (38%)	1.18	0.54–2.58	0.68
**Contaminated urine cultures** ^**b**^	25/56 (45%)	16/53 (30%)	0.54	0.24–1.18	0.12
**Urine cultures with pathogen** ^**c**^	12/56 (21%)	17/53 (32%)	1.73	0.73–4.09	0.21
Colonizations	6/53 (11%)	8/51 (16%)	1.46	0.47–4.54	0.52
Infections^d^	6/53 (11%)	8/51 (16%)	1.46	0.47–4.54	0.52
**Persistence of initial pathogen** ^**c**^	3/56 (5%)	4/51 (8%)	1.50	0.32–7.07	0.61
Persistent colonization	0	1/51 (2%)			
Persistent infection^d^	3/53 (6%)	3/51 (6%)	1.04	0.20–5.42	0.96
**New organism** ^**c**^	9/56 (16%)	11/51 (22%)	1.44	0.54–3.81	0.47
Colonizations	6/53 (11%)	7/49 (14%)	1.31	0.41–4.19	0.65
Infections^d^	3/53 (6%)	3/49 (6%)	1.09	0.21–5.66	0.92
**n (%)**	**Control Group**	**PCT/ Pyuria Group**			
**Total no. of isolates**	13	18			
**Gram-negative uropathogen**					
*Escherichia coli*	3/13 (23%)	3/18 (17%)			
*Klebsiella pneumoniae*	1/13 (8%)	1/18 (6%)			
**Gram-positive uropathogen**					
***Enterococcus faecalis***	**6/13 (46%)**	**11/18 (61%)**			
*Enterococcus faecium*	1/13 (8%)	1/18 (6%)			
*Staphylococcus saprophyticus*	1/13 (8%)	0			
**Non uropathogen**					
Lactobacillus species^b^	1/13 (8%)	1/18 (6%)			
*Candida albicans*	0	1/18 (6%)			

^a^Logistic regression analysis.

^b^Lactobacillus species were considered as contaminants.

^c^In case of contaminated (n = 1 in PCT/Pyuria group) initial urine culture any uropathogen was considered as new organism, in case of missing (n = 2 in PCT/ Pyuria group) initial urine culture the uropathogen was not attributed to persistent or new organism group.

^d^Infection was assumed if pyuria (>20 leukocytes/μL) was present in concomitant urinalysis.

The rate of persistent symptomatic infection 30 days after enrollment was not significantly different between the PCT-pyuria group and the control group (4% in both groups, OR, 1.07; 95% CI, 0.14–7.90; *P* = 0.95 in ITT analysis). There were no symptomatic infections with new organisms 30 days after enrollment (Table 7).

**Table 7:** **Microbiological and clinical outcome 30 days after enrollment**

**n (%)**	**Control group**	**PCT/Pyuria group**	**Odds ratio**	**95% CI**	***P*** **value** ^**a**^
**All patients (intention-to-treat)**	**(n = 64)**	**(n = 61)**			
**Missing urinalysis**	10/64 (16%)	9/61 (13%)	0.93	0.35–2.48	0.89
**Missing urine cultures**	14/64 (22%)	11/61 (18%)	0.79	0.33–1.90	0.59
**Sterile urine cultures**	19/50 (38%)	8/50 (16%)	0.31	0.12–0.80	**0.02**
**Contaminated urine cultures** ^**b**^	20/50 (40%)	26/50 (52%)	1.63	0.74–3.59	0.23
**Urine cultures with pathogen** ^**c**^	11/50 (22%)	16/50 (32%)	1.67	0.68–4.08	0.26
Colonizations	3/50 (6%)	3/49 (6%)	1.02	0.20–5.33	0.98
Infections^d^	8/50 (16%)	13/49 (27%)	1.90	0.71–5.09	0.20
Symptomatic infections	2/50 (4%)	3/49 (6%)	1.57	0.25–9.80	0.63
**Persistence of initial pathogen** ^**c**^	6/50 (12%)	8/48 (17%)	1.47	0.47–4.59	0.51
Persistent colonizations	1/50 (2%)	1/47 (2%)	1.07	0.06–17.53	0.97
Persistent infections^d^	5/50 (10%)	7/47 (15%)	1.58	0.46–5.36	0.47
Persistent symptomatic infections^d^	2/50 (4%)	2/47 (4%)	1.07	0.14–7.90	0.95
**New organism** ^**c**^	5/50 (10%)	6/48 (13%)	1.29	0.37–4.53	0.70
Colonizations	2/50 (4%)	2/47 (4%)	1.07	0.14–7.90	0.95
Infections^d^	3/50 (6%)	4/47 (9%)	1.46	0.31–6.89	0.64
Symptomatic infections^d^	0	0			
**n (%)**	**Control group**	**PCT/Pyuria group**			
**Total no. of isolates** ^**e**^	17	22			
**Gram-negative uropathogen**					
*Escherichia coli*	5/17 (29%)	7/22 (32%)			
*Klebsiella pneumoniae*	2/17 (12%)	1/22 (5%)			
*Citrobacter koseri*	1/17 (6%)	1/22 (5%)			
*Enterobacter cloacae*	0	1/22 (5%)			
*Pseudomonas aeruginosa*	0	1/22 (5%)			
**Gram-positive uropathogen**					
***Enterococcus faecalis***	**5/17 (29%)**	**9/22 (41%)**			
*Enterococcus faecium*	1/17 (6%)	0			
*Staphylococcus saprophyticus*	0	1/22 (5%)			
Group B Streptococci	1/17 (6%)	0			
**Non-uropathogen**					
*Staphylococcus aureus*	1/17 (6%)	0			
Coagulase-negative staphylococci^b^	0	1/22 (5%)			
Lactobacillus species^b^	1/17 (6%)	1/22 (5%)			
*Streptococcus milleri*	0	1/22 (5%)			

^a^Logistic regression analysis.

^b^Lactobacillus species and Coagulase-negative staphylococci were considered as contaminants.

^c^In case of missing initial urine culture (n = 2 in the PCT/Pyuria group) any uropathogen was not attributed to persistent or new organism group.

^d^Infection was assumed if pyuria (>20 leukocytes/μL) was present in concomitant urinalysis.

^e^Two isolates in nine cultures.

### Bacteremic patients

As the distribution of patients with bacteremia was not well balanced between the two study arms we performed a stratified analysis of the primary and secondary outcomes for bacteremic and non-bacteremic patients (Additional file 1: Table S1). As a sensitivity analysis for the primary endpoint we also performed median regression analysis adjusted for bacteremia. Adjusted results were robust and showed a significant reduction in antibiotic exposure (coefficient, −3.0; 95% CI, −0.6 to −5.4; *P* = 0.015). Among bacteremic patients, the initial antibiotic therapy was shorter in the PCT-pyuria group (7.0 vs. 11.0 days, *P* ≤0.001); overall antibiotic exposure within 90 days was not different. The recurrence rate was higher in the PCT-pyuria group (56% vs. 16% in the control group, OR, 6.67; 95% CI, 0.04–1.10; *P* = 0.039) but rehospitalization rates were not significantly different.

The rates of persistent infection 7 days after end of therapy were similar in both groups (13% in the PCT-pyuria group vs. 6% in the control group, OR, 2.14; 95% CI, 0.12–39.47; *P* = 0.61).

There were two symptomatic infections with new organisms 7 days after end of therapy and one persistent symptomatic infection 30 days after enrollment in the control group.

## Discussion

As UTIs are one of the most common indications for antibiotic therapy, the impact of a reduction of treatment duration could be enormous. This study provides evidence that guidance of antibiotic therapy in patients with a UTI by a PCT-pyuria algorithm for antibiotic guidance is feasible. The implementation of an algorithm into clinical workflows is practicable as shown by our group in respiratory tract infections [34]. The efficacy of this approach is evidenced by the 90 day observation period for the primary endpoint, which included both the initial and any subsequent antibiotic treatments.

Overall and in most subgroups (except for the small subgroup of patients with uncomplicated simple UTIs), the PCT-pyuria algorithm led to shorter initial duration of antibiotic therapy. The tested algorithm can help to determine the optimal length of antibiotic therapy and avoid antibiotic overuse.

There was an imbalance in randomization of eight patients with uncomplicated simple UTI with only two being randomized to the PCT-pyuria group. Since all six patients in the standard guidelines group were treated according to our algorithm with single-dose fosfomycin, the median duration of the initial antibiotic therapy in this subgroup was similar between both groups.

Only one patient in the PCT-pyuria group, who relapsed with urosepsis and had to be hospitalized for 3 days and treated with intravenous antibiotics, was initially treated with NSAIDs according to the study protocol. Given the small number of outpatients, the safety of NSAIDs for simple uncomplicated UTIs cannot be determined based on our study. Despite recent data with promising results for this approach [32,33], a recent review [13] emphasized the need for immediate antimicrobial therapy rather than delayed treatment or symptomatic management with ibuprofen alone.

Patients with complicated febrile UTI were older and had multiple comorbidities. Especially in this population with the highest disease burden, in whom the longest antibiotic therapy is recommended by current guidelines, our algorithm proved to be effective.

The safety subgroups were small, but no trend for difference in rates of persistent infection was shown at 7 days after end of therapy or at 30 days after enrollment. The slightly higher, but non-significant, rate of bacteriuria and asymptomatic infection in the PCT-pyuria group should not be viewed as an unsuccessful treatment. There is vast evidence that asymptomatic bacteriuria should not be treated [35]. In fact, asymptomatic bacteriuria might be protective against future development of pyelonephritis, as elegantly shown in a prospective Italian study of young women with asymptomatic bacteriuria, who benefited from symptomatic treatment versus antibiotics [36]. There is evidence that in older patients with isolated non-specific signs or non-infectious symptoms, such as delirium, urine testing and subsequent antibiotic therapy should not be ordered [37].

Furthermore, the follow-up mid-stream urine cultures in the PCT-pyuria group yielded in the majority of cases *Enterococcus faecalis* (Table 2), which has recently been shown to have a low positive predictive value for uropathogen growth in the bladder [38].

The antibiotics chosen in our algorithm, derived from international guidelines, should not deter from the necessity to adapt antibiotic choices to local epidemiology and resistance data [9]. In Switzerland, 18.5% of *Escherichia coli* were resistant to fluoroquinolones in 2013, compared to 22.3% in the European Union in 2012 [39,40].

A major strength was the innovative study design with antibiotic guidance based on the combination of the systemic and local response against a control group receiving antibiotic therapy according to the low end of currently recommended guidelines.

### Potential limitations

Our study has the following limitations. First, the small sample size due to slow recruitment and the heterogeneity of patients within the predefined subgroups limit the power of the analysis, especially regarding safety outcomes. Second, the follow-up mode by telephone interview and by urine samples which were returned by mail harbors potential recall bias and underreporting, which we tried to counteract by contacting the primary care physician and by obtaining discharge letters of subsequent hospitalizations. Third, the analysis of the recurrence rate in the subgroup of patients with bacteremia was limited due to the small numbers. Further studies will have to clarify whether this algorithm needs to be adapted for patients with bacteremia.

## Conclusions

An algorithm based on PCT and pyuria significantly reduces antibiotic exposure when compared to current guidelines without apparent negative effects on clinical outcomes, especially in patients with complicated febrile UTI. Further randomized controlled multi-center studies with larger patient numbers are needed to confirm our findings for all subgroups and to proof the feasibility in other settings.

## Additional file

Additional file 1: Table S1. Analysis stratified for presence of bacteremia.

## Abbreviations

eGFR: Estimated glomerular filtration rate
ITT: Intention-to-treat
NSAIDs: Non-steroidal anti-inflammatory drugs
PCT: Procalcitonin
PP: Per-protocol
UTIs: Urinary tract infections.
